# Combined First-Trimester PAPP-A and Free β-hCG Levels for the Early Diagnosis of Placenta Accreta Spectrum and Placenta Previa: A Case-Control Study

**DOI:** 10.3390/ijms26136187

**Published:** 2025-06-27

**Authors:** Vera Belousova, Irina Ignatko, Irina Bogomazova, Evdokiya Zarova, Svetlana Pesegova, Anastasia Samusevich, Madina Kardanova, Oxana Skorobogatova, Tatiana Kuzmina, Natalia Kireeva, Anna Maltseva

**Affiliations:** I.M. Sechenov First Moscow State Medical University (Sechenov University), 119991 Moscow, Russiabogomazova_i_m@staff.sechenov.ru (I.B.); zarovaea@mail.ru (E.Z.); pesegova_s_v@staff.sechenov.ru (S.P.); samusevich_a_n@staff.sechenov.ru (A.S.); kardanova_m_a@staff.sechenos.ru (M.K.); aisha27_sum@mail.ru (O.S.); kuzmina_t_e@staff.sechenov.ru (T.K.); kireeva_n_v_1@staff.sechenov.ru (N.K.); maltseva_a_g@staff.sechenov.ru (A.M.)

**Keywords:** placenta accreta spectrum, placenta previa, placenta increta, placenta percreta, biomarkers, pregnancy-associated plasma protein-A, free beta subunit of human chorionic gonadotropin

## Abstract

Placenta accreta spectrum (PAS) and placenta previa (PP) are severe obstetric disorders associated with high maternal and perinatal morbidity. Early diagnosis of both conditions remains challenging, particularly in cases with subtle imaging findings. This study was aimed to evaluate the diagnostic value of first-trimester maternal serum levels of pregnancy-associated plasma protein-A (PAPP-A) and free beta subunit of human chorionic gonadotropin (β-hCG) in predicting PAS and PP. In this retrospective case–control study, a total of 100 pregnant women were included: 36 with PAS, 32 with PP, and 32 healthy controls. Serum levels were measured at 11–13^6^ weeks of gestation. Both biomarkers were significantly altered in pathological groups compared to controls: PAPP-A was lower in PP (3.04 [1.42–4.52] IU/L) and PAS (3.63 [2.51–5.39] IU/L) vs. controls (5.34 [3.72–8.41] IU/L; *p* < 0.001), while β-hCG was higher in PP (45.4 [40.1–54.9] IU/L) and PAS (51.4 [32.3–74.8] IU/L) vs. controls (33.5 [22.7–54.1] IU/L; *p* = 0.044 and *p* < 0.001, respectively). ROC analysis demonstrated that combined biomarker modeling improved diagnostic accuracy over single-marker use, with AUCs reaching 0.85 (sensitivity 85.2%, specificity 72%) for PAS and 0.88 (sensitivity 100%, specificity 72%) for PP. These findings support the integration of biochemical screening into first-trimester risk assessment protocols. Incorporating maternal serum biomarkers may enhance early identification of high-risk pregnancies, allow timely referral to specialized care, and reduce adverse outcomes. Further prospective studies are warranted to validate the utility of this dual-marker approach across diverse populations and clinical settings.

## 1. Introduction

Placenta accreta spectrum (PAS) is a severe pregnancy complication in which chorionic villi partially or completely invade the myometrium. In such cases spontaneous placental separation does not occur despite uterotonic therapy and attempts at manual removal may lead to massive hemorrhage [1,2,3]. Depending on the depth of invasion, several variants of this pathology are placenta accreta, increta and percreta [4,5]. Currently, there is a widespread increase in the prevalence of this pathology, because of the rising number of cases of cesarean deliveries, as a uterine scar is the main risk factor of abnormal placental attachment. Other risk factors are intrauterine medical interventions, inflammatory processes, uterine malformations, adenomyosis, and other uterine pathologies [6].

The diagnosis of placenta accreta remains a highly relevant issue. It is important not only to detect abnormal placental attachment but also to determine the depth of invasion, as these factors define management strategies to prevent severe postpartum complications. Ultrasound imaging is the primary diagnostic step for PAS. However, despite diagnostic protocols, this method remains subjective and heavily depends on the operator’s expertise [7,8].

Magnetic resonance imaging (MRI) is a more accurate method for PAS assessment, but it is less accessible, more expensive, and not part of routine practice [9]. MRI is usually performed after ultrasound to confirm pathological placental attachment [10]. Due to these limitations, the search for some biomarkers of PAS is very important.

A number of biomarkers are used to assess the risk of great obstetrical syndromes: fetal growth restriction, preeclampsia, preterm labor. These pathologies are associated with insufficient trophoblast invasion [11]. The pathogenesis of placenta accreta spectrum (PAS) remains incompletely understood; however, the excessive invasion of trophoblast cells into the maternal decidua plays a crucial role. This shared pathophysiological basis (dysregulation of trophoblast invasion) suggests that established early-pregnancy biomarkers of placentation, in particular pregnancy-associated plasma protein-A (PAPP-A) and free beta subunit of human chorionic gonadotropin (free β-HCG), could be markers of abnormal invasion patterns in PAS [12,13,14]. Moreover, accumulating evidence suggests that low levels of PAPP-A and free β-hCG are associated not only with abnormal placentation but also with preterm labor and adverse neonatal outcomes [15,16,17]. These associations highlight their potential value for early identification of high-risk pregnancies, including those complicated by PAS [14,18].

In this study, we assessed and compared the basic clinical characteristics, diagnostic methods, and level of PAPP-A and free β-hCG during the first trimester in women with PAS, non-adherent placenta previa (PP), and healthy controls. We assessed the sensitivity and specificity of these markers for PAS and PP diagnosis independently. In addition, our study represents the first assessment of the combined diagnostic utility of PAPP-A and β-hCG.

## 2. Results

### 2.1. Baseline Characteristics and Clinical History of the Three Groups

A total of 100 patients were included in the assessment of baseline characteristics: 32 healthy controls, 32 women with non-adherent PP and 36 women with PAS. The clinical history of all groups is presented in Table 1.

The study groups were homogeneous in age and BMI (*p* > 0.05), ensuring comparable baseline characteristics. No significant intergroup differences were observed in the history of isolated intrauterine operations (IUO) (curettage, vacuum aspiration) (*p* = 0.68). The PAS cohort demonstrated a substantially higher prevalence of prior cesarean sections (CS) (91.67% vs. 28.13% in controls; *p* < 0.0001). Significantly, the coexistence of prior intrauterine operations and CS was associated with the highest PAS incidence (52.78% vs. 15.63% in controls; *p* < 0.0001). Moreover, our findings reveal a significant prevalence of patients with one prior CS in the PAS group, establishing single CS as an important risk factor for placenta accreta (41.67% vs. 12.5% in controls, *p* = 0.015). However, the lack of a statistically significant difference among patients with three or more previous CSs might be attributed to the limited sample size. Beyond the primary associations, the PAS cohort exhibited elevated parity frequency, underscoring its link to multiparity (*p* < 0.0001).

### 2.2. Pregnancy Outcomes

Table 2 presents critical obstetric and surgical outcomes comparing three patient groups. PAS cases had the most severe clinical profile, with significantly earlier gestational age at delivery (median 35.1 weeks, *p* < 0.0001). This corresponds to Russian clinical guidelines, which recommend delivery at 34–36 weeks in PAS to reduce the risk of massive hemorrhage. In PAS group there are the highest rates of preterm birth (77.8%, *p* < 0.0001) and greatest intraoperative blood loss (median 2500 mL, *p* < 0.0001) compared to controls. The PAS group required the additional interventions to stop hemorrhage more frequently: uterine artery ligation (52.78%, *p* < 0.0001) and endovascular embolization (47.22%, *p* < 0.0001). Placenta previa cases showed intermediate severity, with higher preterm birth rates (34.4%, *p* = 0.009) and blood loss (750 mL, *p* < 0.0001) than controls but lower rates than PAS. Neonatal outcomes followed a similar gradient, with PAS associated with the highest rates of hypoxia (75% overall, *p* < 0.0001) and lowest birth weights (median 2600 g, *p* < 0.0001). The control group consistently showed the most favorable outcomes across all measured parameters. These findings demonstrate a clear clinical spectrum from normal placentation to placenta previa to PAS, with progressively worsening maternal and neonatal outcomes.

### 2.3. Diagnostic Methods for PAS

**Figure 1:**
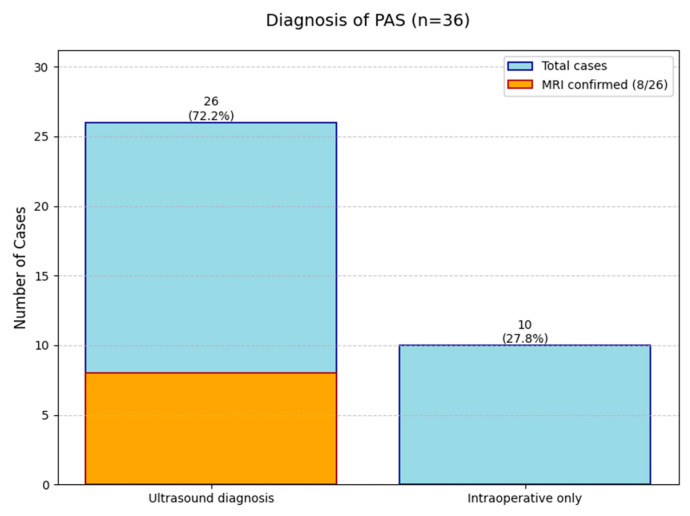
The diagnostic outcomes for 36 Placenta Accreta Spectrum (PAS) cases.

To assess the current efficiency of PAS diagnostic approaches, we analyzed clinical data from a cohort of 36 patients (Table 3). Ultrasound examination with typical signs served as the primary diagnostic method in 26 cases (72.2% of total cases, 95% CI: 55.9–84.2%). Among these ultrasound-detected cases, MRI confirmation was obtained in eight patients (representing 30.8% of ultrasound-positive cases and 22.2% of the entire cohort, 95% CI: 11.8–38.1%). The majority were diagnosed by ultrasound without MRI confirmation (18 cases, 69.2% of ultrasound group and 50.0% of total). A total of 10 cases were identified only during surgical intervention, highlighting the limitations of preoperative diagnostic methods (27.8% of total, 95% CI: 15.7–44.1%) (Figure 1).

The data reveals that while ultrasound is the main diagnostic tool (successful in 72% of cases), MRI plays a complementary role in confirming about one-third of ultrasound-positive cases. However, a significant number of PAS cases (28%) remain undetectable until surgery. This analysis underscores the importance of a multimodal diagnostic approach while identifying opportunities for optimization in current PAS diagnostic pathways.

### 2.4. Biomarkers

The final analysis of serum levels of PAPP-A and β-hCG were compared across three groups: healthy controls (*n* = 32), PP (*n* = 22), and PAS (*n* = 27). The reduction from the initial cohort appeared due to missing laboratory measurements.

Median PAPP-A levels were significantly lower in both PP and PAS groups compared to controls (control vs. PP: *p* < 0.001; control vs. PAS: *p* < 0.001). No significant difference was observed between the PP and PAS groups (*p* = 0.891), which may be because of the common pathophysiological mechanism. On the contrary, free β-hCG levels were significantly higher in both PAS (*p* < 0.001) and PP (*p* = 0.044) compared to controls, with a greater increase in PAS. However, this difference between PAS and PP did not reach statistical significance (*p* = 0.318) (Table 4).

**Figure 2:**
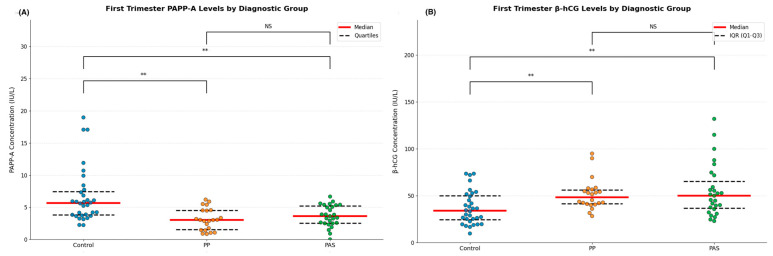
(**A**) Distribution of individual measurements of PAPP-A with median (red line) and interquartile range (dashed black lines) for healthy controls (*n* = 32), placenta previa (PP, *n* = 22), and placenta accreta spectrum (PAS, *n* = 27) groups. ** *p* < 0.001; NS = not significant. (**B**) Distribution of individual measurements of free β-HCG with median (red line) and interquartile range (dashed black lines) for healthy controls (*n* = 32), placenta previa (PP, *n* = 22), and placenta accreta spectrum (PAS, *n* = 27) groups. ** *p* < 0.001; NS = not significant.

These data show significant alterations in first-trimester PAPP-A and β-hCG levels in pregnancies with placental pathologies. While both markers effectively differentiate pathological from healthy pregnancies, they show limited utility in distinguishing between PAS and PP. Biomarker distributions across the three groups are visualized in Figure 2.

Considering the observed differences in serum biomarker levels among the study groups, we additionally conducted a ROC analysis to evaluate the diagnostic performance of PAPP-A and β-hCG—individually and in combination to figure out placental pathological invasion from normal pregnancies.

The ROC analysis showed PAPP-A levels provide high diagnostic efficiency to identify PP and PAS. For PP, the analysis yielded an AUC of 0.815 (95% CI: 0.68–0.92), with an optimal threshold of 3.25 IU/L, achieving a sensitivity of 63.6% and specificity of 90.6%. In comparison, PAS showed a slightly lower AUC of 0.752 (95% CI: 0.62–0.86), with an optimal cutoff value of 5.59 IU/L, sensitivity of 92.6%, and specificity of 53.1%. The cutoff points were determined using the Youden index to maximize the combined sensitivity and specificity.

**Figure 3:**
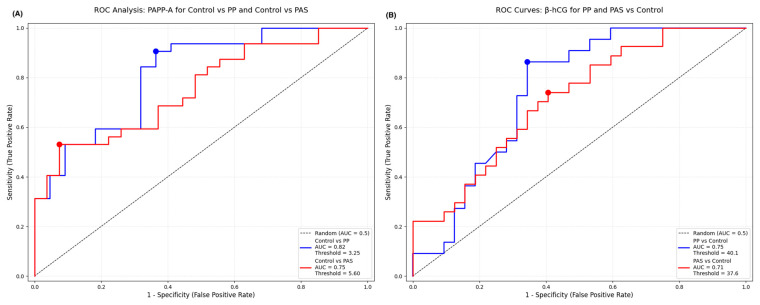
(**A**) ROC curve for PAPP-A distinguishing placenta previa (PP) and placenta accreta spectrum (PAS) from healthy controls. The graph shows AUC values, optimal cutoff points, sensitivity, and specificity for each comparison. (**B**) ROC curve for β-hCG levels in the first trimester for identifying PP and PAS cases versus controls. Diagnostic performance is summarized with AUC, sensitivity, and specificity.

These data suggest that while PAPP-A demonstrates high specificity for PP, it offers higher sensitivity in the context of PAS, which may support its utility as a screening biomarker for placental abnormalities. The corresponding ROC curves with annotated thresholds are presented in Figure 3A.

The diagnostic significance of free β-hCG levels was also evaluated using ROC curve analysis to differentiate both PP and PAS cases from healthy controls. For PP the AUC was 0.748 (95% CI: 0.61–0.87), with an optimal cutoff value of 40.1 IU/L, yielding a sensitivity of 86% and specificity of 66%. In the PAS group, the AUC was 0.713 (95% CI: 0.58–0.83), with an optimal threshold of 37.6 IU/L, achieving 74% sensitivity and 59% specificity.

These data suggest higher β-hCG levels may assist in the early identification of placental pathologies with particularly high sensitivity in PP cases. But the modest specificity, especially for PAS, highlights the need for caution when using β-hCG as a standalone screening tool. The ROC curves with corresponding cutoff points are shown in Figure 3B.

### 2.5. Combined Marker Analysis

Considering the individual diagnostic value of PAPP-A and β-hCG, we further evaluated their combined utility for distinguishing pathological pregnancies. Separate analyses were conducted for PAS and PP subgroups. Table 5 presents the diagnostic performance of PAPP-A and free β-hCG as individual and combined biomarkers.

To account for the different expression—specifically, the lower levels of PAPP-A in pathological cases—PAPP-A values were inverted and both markers were min–max normalized. Logistic regression models incorporating both normalized PAPP-A and β-hCG values were developed for each subgroup. Logistic regression was chosen due to its suitability for binary classification and interpretability in clinical practice. Predicted probabilities were subsequently used for ROC curve construction and AUC estimation to quantify the added value of the combined marker approach.

**Figure 4:**
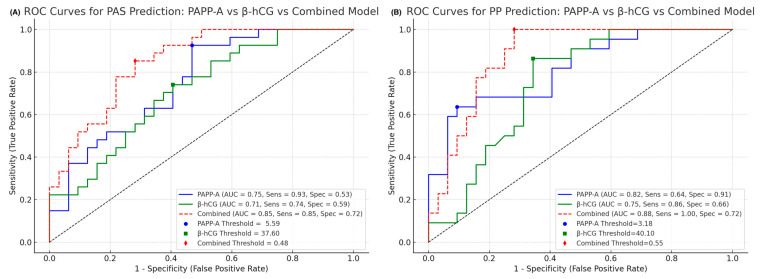
(**A**) ROC curve for the combined model of normalized PAPP-A and β-hCG levels in the first trimester for distinguishing placenta accreta spectrum (PAS) cases from controls. The model demonstrates improved diagnostic accuracy, with performance summarized by AUC, sensitivity, and specificity. (**B**) ROC curve for the combined model of normalized PAPP-A and β-hCG levels in the first trimester for identifying postpartum preeclampsia (PP) cases versus controls. Diagnostic performance is evaluated using the area under the curve (AUC), with corresponding sensitivity and specificity values provided.

In the PAS group, the combined model achieved an area under the ROC curve (AUC) of 0.85, indicating improved discriminatory ability compared to either marker alone (PAPP-A: 0.75; β-hCG: 0.71) (Figure 4A).

Similarly, in the PP group, the combined model yielded an AUC of 0.88, again outperforming individual marker analyses (PAPP-A: 0.82; β-hCG: 0.75) (Figure 4B).

## 3. Discussion

Placenta accreta spectrum (PAS) remains one of the most serious obstetric problems for its negative effect on both maternal and neonatal complications. Our study shows that PAS is associated with severe intraoperative complications: life-threatening hemorrhage (median blood loss 2500 mL) and essential blood transfusion, high risk of bladder injury, and hysterectomy. Due to preterm delivery, neonatal outcomes in PAS cases are also problematic: low birth weights (median weight 2600 g [580–3630]) and high rates of neonatal hypoxia (75%). These data are consistent with the recent literature documenting the severe morbidity associated with PAS [19].

Early prenatal diagnosis of placenta accreta is critically important as it facilitates three essential management strategies: (1) planned transfer to specialized facilities with multidisciplinary teams; (2) coordinated surgical planning to minimize organ injuries; and (3) proactive neonatal intensive care unit engagement to improve neonatal outcomes [6].

Ultrasound remains the first-line diagnostic option to detect placenta accreta spectrum disorders. The study by J. Panaiotova et al. (2019) revealed that the assessment of first-trimester sonographic markers in high-risk pregnancies (women with low-lying placenta and a history of uterine surgery) provided high prognostic accuracy for subsequent PAS diagnosis [8]. The systematic review of F. D’Antonio et al. (2013) demonstrates that ultrasonography has high diagnostic accuracy for placenta accreta spectrum disorders, with a pooled sensitivity of 90.7% and specificity approaching 97% [20]. Doppler ultrasonography shows particular diagnostic value, maintaining 90.7% sensitivity while providing 87.7% specificity for invasion detection [20]. However, the diagnostic performance of ultrasound significantly depends on operator experience, technical factors (e.g., BMI, posterior placental location), and anatomical variations (such as limited myometrial visualization in early gestation and bowel gas interference). These factors also introduce significant confounding in the evaluation and comparison of study results, making it challenging to accurately assess the diagnostic efficiency of this method.

MRI has emerged as a more precise diagnostic modality for evaluating the topography and depth of placental invasion, particularly in cases of posterior placental location. It demonstrates remarkable diagnostic performance across PAS subtypes with near-perfect sensitivity for placenta increta (100%; 95% CI 75.3–100) and high sensitivity for both accreta (94.4%; 95% CI 15.8–99.9) and percreta (86.5%; 95% CI 74.2–94.4) [9].

Despite these compelling accuracy metrics, clinical implementation faces substantial barriers, as evidenced by our institutional data showing only 22.2% (8/36) of high-risk patients received MRI evaluation. Most concerning was the 27.8% (10/36) of cases where ultrasound failed to detect PAS, resulting in intraoperative PAS diagnosis—a scenario associated with significantly worse maternal outcomes [7,8].

This diagnostic gap underscores the urgent need for improved risk stratification. We propose to complete the current diagnostic approach of PAS with the first-trimester biochemical markers (PAPP-A and β-hCG) routinely measured during the first screening [21,22]. These serum biomarkers may enhance early identification of high-risk PAS pregnancies and potentially reduce the false-negative rate observed in ultrasound screening alone.

PAPP-A (pregnancy-associated plasma protein A)—a zinc-dependent metalloproteinase produced by the syncytiotrophoblast. PAPP-A—plays a role in the proteolysis of IGFBP-4 (insulin-like growth factor-binding protein 4), thereby modulating the bioavailability of IGFs (insulin-like growth factors), which participate in regulating of various processes involved in trophoblast invasion, although their precise role remains unclear [22]. Currently, this biomarker is used to assess the risk of fetal chromosomal abnormalities, preeclampsia, fetal growth restriction, preterm birth, pregnancy loss, and low birth weight [13].

Free β-hCG is the earliest detectable glycoprotein in maternal circulation, produced by the syncytiotrophoblast to support early pregnancy through progesterone regulation, angiogenesis, trophoblast differentiation and immune modulation [11,23]. Despite its key role in placental development, studies on its association with PAS remain inconclusive, likely due to high variability in first-trimester levels and overlaps with other pathologies [11,23]. Given its limited specificity as a standalone marker, combining β-hCG with other biomarkers may enhance early detection of PAS and improve diagnostic accuracy in high-risk groups.

Our data demonstrate that first-trimester maternal serum levels of PAPP-A and free β-hCG are significantly altered in pregnancies complicated by placental pathologies, including PAS and PP. Specifically, we found that median PAPP-A concentrations were significantly lower in both PP and PAS groups compared to healthy controls (controls: 5.34 [3.72–8.41] vs. PP 3.04 [1.42–4.52], *p* < 0.001; vs. PAS 3.63 [2.51–5.39], *p* < 0.001) This reduction supports the hypothesis of early placental dysfunction common to both conditions. The lack of a significant difference between the PP and PAS groups further suggests a shared pathophysiological pathway, possibly rooted in abnormal trophoblast invasion. (PP vs. PAS: *p* = 0.891).

However, according to a recent systematic review by Y. Li et al. (2023), which included 243 women with PAS, first-trimester PAPP-A levels were significantly higher in women with PAS (mean difference: 0.43 MoM, 95% CI [0.30 to 0.56], *p* < 0.001; I2 = 32%); our data demonstrated significantly lower concentrations in the PAS group compared to controls [24]. This discrepancy may reflect differences in study design, population characteristics, or diagnostic criteria. These findings highlight the heterogeneity of placental invasion disorders and underscore the need for further research to clarify the biological behavior of PAPP-A in early pregnancy across PAS subtypes.

Conversely, free β-hCG levels were significantly elevated in both PAS and PP cases compared to controls with a more marked increase observed in PAS. (controls: 33.5 [22.7–54.1] vs. PP: 45.4 [40.1–54.9], *p* = 0.044; vs. PAS: 51.4 [32.3–74.8], *p* < 0.001). Although the difference in β-hCG levels between PAS and PP was not statistically significant (PP vs. PAS: *p* = 0.318), some reports have also noted heightened β-hCG in pregnancies with abnormal placental invasion, potentially reflecting exaggerated trophoblastic activity.

A key strength and unique contribution of this study is the combined evaluation of PAPP-A and β-hCG as a multivariable diagnostic model. While previous studies have primarily examined these markers individually, our approach integrates both biomarkers into a unified logistic regression model, accounting for their distinct but complementary expression patterns. This combination significantly improved diagnostic performance, yielding higher AUC values and better sensitivity/specificity profiles than either marker alone. These findings highlight the clinical potential of using a combined biomarker strategy in first-trimester screening protocols to early identification of pregnancies at risk for PAS or PP.

Still, our study has several limitations. First, the relatively small sample size may limit the ability to generalize the findings, increase the risk of selection bias, and does not allow for a formal a priori sample size calculation. The limited number of PAS and PP cases restricts the statistical power to detect subtle differences between groups and may affect the stability of the combined biomarker models. Additionally, as this was a retrospective, single-center study, unmeasured confounding factors cannot be entirely excluded. Hence, further research involving larger, multi-center cohorts is essential to validate our findings and confirm the utility of combined first-trimester markers in predicting placental pathologies.

Our study proposes that combining PAPP-A with free β-hCG may improve the early detection of placenta accreta spectrum (PAS) disorders. This dual-marker approach, potentially integrated with established ultrasound criteria, may offer improved risk stratification for PAS in high-risk pregnancies while maintaining clinically acceptable specificity. Authors should discuss the results and how they can be interpreted from the perspective of previous studies and of the working hypotheses. The findings and their implications should be discussed in the broadest context possible. Future research directions may also be highlighted.

## 4. Materials and Methods

### 4.1. Study Design and Population

A retrospective case–control study was performed over a 3-year period (1 January 2022–31 December 2024). The inclusion criteria were as follows: (1) PAS group: women with a confirmed diagnosis of placenta accreta spectrum by intraoperative findings and histopathological verification; (2) placenta previa group (PP): women with a confirmed diagnosis of placenta previa as determined by intraoperative findings and histopathological verification; (3) control group: women without placental pathology and its normal location determined by clinical findings. The exclusion criteria: (1) twin or multiple pregnancy; (2) miscarriages or stillbirths; (3) data missing or outliers of PAPP-A and free beta-HCG.

**Figure 5:**
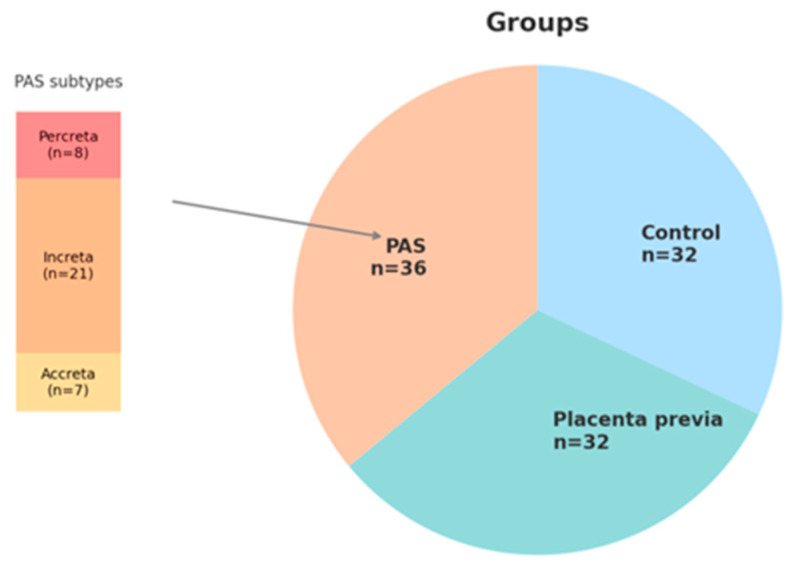
Distribution of study participants across clinical groups and detailed classification of PAS cases.

A total of 100 patients were included in the study: 36 in the PAS group (7 placenta accreta, 21 placenta increta and 8 placenta percreta), 32 in the non-adherent placenta previa group (PP), and 32 in the healthy control group. While all enrolled patients were included in the analysis of clinical characteristics, biomarker assessment (PAPP-A and free β-hCG) was restricted to subgroups with complete data: 27 PAS cases, 22 PP cases, and 32 healthy controls. The distribution of study participants across clinical groups is presented in Figure 5. All these women were tested for maternal serum PAPP-A and β-HCG level at 11–13^6^ weeks. The data of PAPP-A and free-HCG were obtained directly from the Astraia software 29.2.1 (Astraia software gmbh, Munich, Germany) based on routine first-trimester antenatal screening records. The retrospective design of the study did not require any informed consent and approval of the Ethical Committee of the institution. To protect patient privacy, all data were anonymized.

### 4.2. Statistical Analysis

Continuous variables are presented as mean ± SD (normally distributed) or median [Min–Max] (non-normally distributed), assessed using Shapiro–Wilk test. Categorical variables were analyzed using Fisher’s exact test with Bonferroni correction for multiple comparisons. Between-group comparisons of normally distributed continuous variables were performed using one-way ANOVA followed by Tukey’s post hoc test, while non-normally distributed data were analyzed using Kruskal–Wallis test with Dunn-Bonferroni post hoc comparisons. All statistical analyses and data visualizations were conducted using Python (version 3.9.6) with packages including *seaborn*, *statsmodels*, and *matplotlib*. A two-tailed *p*-value < 0.05 was considered statistically significant, with exact values reported throughout except for extreme significance levels (*p* < 0.0001). Biochemical marker analysis was conducted by comparing PAPP-A and free β-hCG levels using the Kruskal–Wallis test, followed by Dunn’s post hoc test with Bonferroni correction. Diagnostic performance was evaluated through receiver operating characteristic (ROC) curve analysis, with optimal cut-off values determined by maximizing Youden’s index. To assess the ability of maternal serum biomarkers to differentiate pathological pregnancies from controls, combined analyses were performed for two subgroups: placenta accreta spectrum (PAS) and postpartum preeclampsia (PP). Biomarker values were Min–Max normalized, with inverted scaling applied to PAPP-A due to its lower expression in affected cases. Separate logistic regression models were constructed for PAS and PP using the normalized PAPP-A and β-hCG values as predictors. The predicted probabilities from each model were used to construct ROC curves and calculate the corresponding area under the curve (AUC) values.

## 5. Conclusions

This study demonstrates that first-trimester serum levels of PAPP-A and free β-hCG are significantly altered in pregnancies complicated by PAS and PP, reflecting early placental dysfunction. Individually, each marker provides moderate diagnostic value, but their combined use significantly enhances the ability to discriminate pathological from healthy pregnancies. This integrative biomarker approach represents a novel and promising tool for early identification of placental disorders. Future studies with larger cohorts are warranted to validate these findings and support the clinical implementation of combined biomarker screening.

## Figures and Tables

**Table 1:** Baseline characteristics and clinical history of the three groups: control group, placenta previa group (PP), placenta accreta spectrum group (PAS).

	Control (*n* = 32)	PP (*n* = 32)	PAS (*n* = 36)	Pairwise Comparisons (*p*-Values)	Overall *p*-Value
Age (years) ^1^	36.43 ± 4.89	35.38 ± 4.96	35.8 ± 4.3	C vs. PP: *p* = 0.72 C vs. PAS: *p* = 0.88 PP vs. PAS: *p* = 0.92	*p* = 0.62
BMI (kg/m^2^) ^1^	27.38 ± 4.59	28.01 ± 5.72	28.2 ± 4.2	C vs. PP: *p* = 0.88 C vs. PAS: *p* = 0.88 PP vs. PAS: *p* = 1.00	*p* = 0.89
Previous IUO ^2^	15 (46.88%)	19 (59.38%)	21 (58.33%)	C vs. PP: *p* = 1.000 C vs. PAS: *p* = 1.000 PP vs. PAS: *p* = 1.000	*p* = 0.520
Previous CS ^2^	9 (28.13%)	9 (28.13%)	33 (91.67%)	C vs. PP: *p* = 1.000 C vs. PAS: *p* < 0.0001 PP vs. PAS: *p* < 0.0001	*p* < 0.0001
1 previous CS ^2^	4 (12.50%)	4 (12.50%)	15 (41.67%)	C vs. PP: *p* = 1.000 C vs. PAS: *p* = 0.015 PP vs. PAS: *p* = 0.015	*p* = 0.009
2 previous CS ^2^	2 (6.25%)	2 (6.25%)	12 (33.33%)	C vs. PP: *p* = 1.000 C vs. PAS: *p* = 0.009 PP vs. PAS: *p* = 0.009	*p* = 0.0012
3 previous CS ^2^	1 (3.13%)	3 (9.38%)	6 (16.67%)	C vs. PP: *p* = 1.000 C vs. PAS: *p* = 0.321 PP vs. PAS: *p* =1.000	*p* = 0.127
Previous IUO + previous CS ^2^	4 (12.50%)	5 (15.63%)	19 (52.78%)	C vs. PP: *p* = 1.000 C vs. PAS: *p* < 0.0001 PP vs. PAS: *p* = 0.002	*p* < 0.0001
Parity ^3^	1 [0–4]	2 [0–7]	3 [1–9]	C vs. PP: *p* = 0.174 C vs. PAS: *p* < 0.0001 PP vs. PAS: *p* = 0.057	*p* < 0.0001

^1^ mean ± SD, normal distribution of data presented as mean ± standard deviation; ^2^ categorical numbers are expressed by number and percentage; ^3^ median, Min–Max, non-normal distribution is presented as median [Min–Max]; categorical variables were analyzed using Fisher’s exact test with Bonferroni correction for multiple comparisons. For continuous variables the normality was assessed by Shapiro–Wilk test. Continuous variables with normal distribution employed the one-way ANOVA with post hoc Tukey test; continuous variables with a non-normal distribution employed the Kruskal–Wallis test with Dunn-Bonferroni post hoc comparisons; BMI = body mass index; IUO = intrauterine operation; CS = cesarean section.

**Table 2:** Pregnancy outcomes in three groups: control group, placenta previa group (PP), placenta accreta spectrum group (PAS).

	Control (*n* = 32)	PP (*n* = 32)	PAS (*n* = 36)	Pairwise Comparisons (*p*-Values)	Overall *p*-Value
Term of delivery ^3^	39.43 [37.2–41.3]	38 [25–41.3]	35.08 [24–40]	C vs. PP: *p* = 0.174 C vs. PAS: *p* < 0.0001 PP vs. PAS: *p* = 0.057	*p* < 0.0001
Preterm birth ^2^	0	11 (34.38%)	28 (77.78%)	C vs. PP: *p* = 0.0018 C vs. PAS: *p* < 0.0001 PP vs. PAS: *p* = 0.0003	*p* < 0.0001
Intraoperative blood loss (mL) ^3^	550 [487–844]	750 [600–1250]	2500 [870–5120]	C vs. PP: *p* < 0.0001 C vs. PAS: *p* < 0.0001 PP vs. PAS: *p* = 0.027	*p* < 0.0001
Hysterectomy ^2^	0	0	1 (2.78%)	C vs. PP: *p* = 1.000 C vs. PAS: *p* = 1.000 PP vs. PAS: *p* = 1.000	*p* = 0.492
Bladder injury ^2^	0	0	10 (27.78%)	C vs. PP: *p* = 1.00 C vs. PAS: *p* < 0.0001 PP vs. PAS: *p* < 0.0001	*p* < 0.0001
Uterine artery ligation ^2^	0	6 (18.75%)	19 (52.78%)	C vs. PP: *p* = 0.0375 C vs. PAS: *p* < 0.0001 PP vs. PAS: *p* = 0.0036	*p* < 0.0001
Endovascular uterine artery embolization ^2^	0	0	17 (47.22%)	C vs. PP: *p* = 1.000 C vs. PAS: <0.0001 PP vs. PAS: <0.0001	*p* < 0.0001
Neonatal weight (g) ^3^	3490 [1900–4240]	2900 [690–3950]	2600 [580–3630]	C vs. PP: *p*= 0.011 C vs. PAS: *p* < 0.0001 PP vs. PAS: *p* = 0.046	*p* < 0.0001
No neonatal hypoxia ^2^	30 (93.75%)	19 (59.38%)	9 (25%)	C vs. PP: *p* = 0.003 C vs. PAS: *p* < 0.0001 PP vs. PAS: *p* = 0.012	*p* < 0.0001
Mild neonatal hypoxia ^2^	2 (6.25%)	10 (31.25%)	21 (58.33%)	C vs. PP: *p* = 0.063 C vs. PAS: *p* < 0.0001 PP vs. PAS: *p* = 0.069	*p* < 0.0001
Moderate neonatal hypoxia ^2^	0	1 (3.13%)	5 (13.89%)	C vs. PP: *p* = 1.000 C vs. PAS: *p* = 0.147 PP vs. PAS: *p* = 0.594	*p* = 0.028
Severe neonatal hypoxia ^2^	0	2 (6.25%)	1 (2.78%)	C vs. PP: *p* = 1.000 C vs. PAS: *p* = 1.000 PP vs. PAS: *p* = 1.000	*p* = 0.593

^2^ categorical numbers are expressed by number and percentage; ^3^ median, Min–Max, non-normal distribution is presented as median [Min–Max]; categorical outcomes were assessed via Fisher’s exact test with Bonferroni correction for multiple comparisons. Continuous variables with non-normal distribution (confirmed by Shapiro–Wilk testing) employed the Kruskal–Wallis test with Dunn’s pairwise comparisons.

**Table 3:** Diagnostic methods for PAS.

Diagnostic Methods	Cases (*n*)	% of Ultrasound Group (*n* = 26)	% of Total Cases (*n* = 36)	95% CI (Total) ^1^
Ultrasound detection (typical signs)	26	100%	72.2%	(55.9–84.2%)
(1) With MRI confirmation	8	30.8%	22.2%	(11.8–38.1%)
(2) Without MRI	18	69.2%	50.0%	(34.6–65.4%)
Intraoperative-only diagnosis	10	- ^2^	27.8%	(15.7–44.1%)

^1^ Percentages were calculated for each diagnostic pathway relative to subgroup and total cohort sizes; 95% confidence intervals for proportions were derived using binomial distribution methods. Group comparisons were based on absolute case numbers and their relative frequencies. ^2^ In all cases where PAS disorder was diagnosed intraoperatively, the condition had not been detected during preoperative ultrasound examinations.

**Table 4:** Comparison of the PAPP-A and free β-hCG level in three groups.

Biomarkers	Control (*n* = 32)	PP (*n* = 22)	PAS (*n* = 27)	*p* Value ^1^
PAPP-A (IU/L)	5.34 [3.72–8.41]	3.04 [1.42–4.52]	3.63 [2.51–5.39]	C vs. PP: *p* < 0.001 C vs. PAS: *p* < 0.001 PP vs. PAS: *p* = 0.891
free β-hCG (IU/L)	33.5 [22.7–54.1]	45.4 [40.1–54.9]	51.4 [32.3–74.8]	C vs. PP: *p* = 0.044 C vs. PAS: *p* < 0.001 PP vs. PAS: *p* = 0.318

^1^ The Shapiro–Wilk test confirmed non-normal distributions (all *p* < 0.05) and Levene’s test indicated the heterogeneity of variances (*p* = 0.0003); group comparisons were performed using the Kruskal–Wallis test followed by Dunn’s post hoc test with Bonferroni adjustment. Data are presented as median [25th; 75th percentile].

**Table 5:** Diagnostic performance of PAPP-A and free β-hCG as individual and combined biomarkers for PP and PAS.

Biomarkers	Groups	AUC (95% CI)	Optimal Cutoff	Sensitivity	Specificity
PAPP-A (IU/L)	Control vs. PAS	0.75 (0.62–0.86)	5.59 IU/L	92.6%	53.1%
	Control vs. PP	0.82 (0.68–0.92)	3.25 IU/L	63.6%	90.6%
free β-hCG (IU/L)	Control vs. PAS	0.71 (0.58–0.83)	37.6 IU/L	74%	59%
	Control vs. PP	0.75 (0.61–0.87)	40.1 IU/L	86%	66%
PAPP-A + free β-hCG	Control vs. PAS	0.85 (0.75–0.93)	0.48 ^1^	85.2%	72%
	Control vs. PP	0.88 (0.78–0.96)	0.55 ^1^	100%	72%

^1^ The cutoff value is expressed as a predicted probability derived from a logistic regression model based on normalized biomarker levels.

## Data Availability

The datasets generated and analyzed during this study are not publicly available due to patient confidentiality restrictions under Russian Federation healthcare data protection laws. Anonymized data supporting the findings may be made available upon reasonable request from the corresponding author, subject to approval by the Ethics Committee of City Clinical Hospital, named after S.S. Yudin.

## Abbreviations

The following abbreviations are used in this manuscript:

PAS	Placenta accreta spectrum
PP	Placenta previa
MRI	Magnetic resonance imaging
PAPP-A	Pregnancy-associated plasma protein A
β-hCG	Beta subunit of human chorionic gonadotropin
BMI	Body mass index
IUO	Intrauterine operations
CS	Cesarean section
ROC	Receiver operating characteristic
AUC	Area under the curve
